# Clinical usefulness of eribulin as first- or second-line chemotherapy for recurrent HER2-negative breast cancer: a randomized phase II study (JBCRG-19)

**DOI:** 10.1007/s10147-021-01920-0

**Published:** 2021-04-23

**Authors:** Kenjiro Aogi, Kenichi Watanabe, Masahiro Kitada, Takafumi Sangai, Shoichiro Ohtani, Tomoyuki Aruga, Hidetoshi Kawaguchi, Tomomi Fujisawa, Shigeto Maeda, Takashi Morimoto, Nobuaki Sato, Shintaro Takao, Satoshi Morita, Norikazu Masuda, Masakazu Toi, Shinji Ohno

**Affiliations:** 1 Department of Breast Oncology, National Hospital Organization Shikoku Cancer Center, Kou 160, Minamiumemoto-machi, Matsuyama, Ehime, 791-0280 Japan; 2 Department of Breast Surgery, National Hospital Organization Hokkaido Cancer Center, Sapporo, Japan; 3 Breast Disease Center, Asahikawa Medical University Hospital, Asahikawa, Japan; 4 Department of Breast Thyroid Surgery, Kitasato University Hospital, Sagamihara, Japan; 5 Department of Breast Surgery, Hiroshima City Hiroshima Citizens Hospital, Hiroshima, Japan; 6 Department of Breast Surgery, Tokyo Metropolitan Cancer and Infectious Diseases Center Komagome Hospital, Tokyo, Japan; 7 Department of Breast Surgery, Matsuyama Red Cross Hospital, Matsuyama, Japan; 8 Department of Breast Oncology, Gunma Prefectural Cancer Center, Ohta, Japan; 9 Department of Surgery, National Hospital Organization Nagasaki Medical Center, Nagasaki, Japan; 10 Department of Breast Surgery, Yao Municipal Hospital, Osaka, Japan; 11 Department of Breast Oncology, Niigata Cancer Center Hospital, Niigata, Japan; 12 Department of Breast Surgery, Hyogo Cancer Center Hospital, Kobe, Japan; 13 Department of Biomedical Statistics and Bioinformatics, Graduate School of Medicine, Kyoto University, Kyoto, Japan; 14 Department of Surgery, Breast Oncology, National Hospital Organization Osaka National Hospital, Osaka, Japan; 15 Department of Breast Surgery, Graduate School of Medicine, Kyoto University, Kyoto, Japan; 16 Breast Oncology Center, The Cancer Institute Hospital of Japanese Foundation for Cancer Research, Tokyo, Japan

**Keywords:** Breast cancer, Eribulin, Clinical trial

## Abstract

**Background:**

Anthracycline (A) or taxane T-based regimens are the standard early-line chemotherapy for metastatic breast cancer (BC). A previous study has shown a survival benefit of eribulin in heavily pretreated advanced/recurrent BC patients. The present study aimed to compare the benefit of eribulin with treatment of physician’s choice (TPC) as first- or second-line chemotherapy for recurrent HER2-negative BC.

**Methods:**

Patients with recurrent HER2-negative BC previously receiving anthracycline and taxane AT-based chemotherapy in the adjuvant or first-line setting were eligible for this open-label, randomized, parallel-group study. Patients were randomized 1:1 by the minimization method to receive either eribulin (1.4 mg/m^2^ on day one and eight of each 21-day cycle) or TPC (paclitaxel, docetaxel, nab-paclitaxel or vinorelbine) until disease progression or unacceptable toxicity. The primary endpoint was progression-free survival (PFS). Secondary endpoints included time to treatment failure (TTF), overall response rate (ORR), duration of response, and safety (UMIN000009886).

**Results:**

Between May 2013 and January 2017, 58 patients were randomized, 57 of whom (26 eribulin and 31 TPC) were analyzed for efficacy. The median PFS was 6.6 months with eribulin versus 4.2 months with TPC (hazard ratio: 0.72 [95% confidence interval (CI), 0.40–1.30], *p* = 0.276). Median TTF was 6.0 months with eribulin versus 3.6 months with TPC (hazard ratio: 0.66 [95% CI, 0.39–1.14], *p* = 0.136). Other endpoints were also similar between groups. The most common grade ≥ 3 adverse event was neutropenia (22.2% with eribulin versus 16.1% with TPC).

**Conclusions:**

Eribulin seemed to improve PFS or TTF compared with TPC without statistical significance. Further validation studies are needed.

## Introduction

Anthracycline (A) or taxane T-based regimens are the standard chemotherapy for patients with HER2-negative breast cancer who have developed recurrent disease after surgery. However, anthracyclines and taxanes (AT) are usually avoided in patients who have already received AT as neoadjuvant/adjuvant therapy [1]. Chemotherapy with paclitaxel/docetaxel (if not used in prior treatment), nab-paclitaxel and vinorelbine is described to be commonly acceptable for HER2-negative metastatic breast cancer (MBC) with prior anthracyclines/taxane, which is described in the Japanese chemotherapy guideline for breast cancer in the first or second treatment line (The Japanese Breast Cancer Society Clinical Practice Guideline for Systemic Treatment of Breast Cancer in Japanese). Although no standard strategy has yet been established for the first choice of the first-line regimen for recurrent disease after surgery, microtubule inhibitors are among promising therapies for advanced recurrent breast cancer.

Eribulin, a fully synthetic analog of halichondrin B isolated from the marine sponge Halichondria okadai, is a non-taxane microtubule dynamics inhibitor that inhibits the elongation (polymerization), but not shortening (depolymerization), of microtubules to induce cancer cell death [2–6]. In the phase III Eisai metastatic breast cancer study assessing physician’s choice versus E7389 (EMBRACE) study conducted outside Japan, eribulin improved overall survival by 2.7 months compared with treatment of physician’s choice (TPC) in patients with advanced recurrent breast cancer who had received two or more previous chemotherapy regimens, including AT. This improvement was also observed in the ER-positive patient subgroup [7]. A Japanese phase II single-arm study also found that eribulin therapy was highly effective and well tolerated in heavily pretreated patients [8]. Eribulin has, thus, been shown to provide a survival benefit in patients with advanced recurrent breast cancer pretreated with chemotherapy, including AT, and therefore, it is meaningful to evaluate its clinical usefulness compared to existing intravenous breast cancer therapies in early-line treatment of recurrent disease after surgery.

With this background, to evaluate the clinical usefulness of eribulin in comparison with TPC as first- or second-line treatment for recurrent HER2-negative breast cancer in patients who had previously received AT containing regimens and to determine whether to proceed to a phase III study, we conducted a phase II study (JBCRG-19) to investigate the superiority of eribulin over TPC for progression-free survival (PFS) as the primary endpoint.

## Patients and methods

This was a multicenter, open-label, randomized phase II study of eribulin versus TPC in patients with recurrent HER2-negative breast cancer conducted at 21 centers in Japan (UMIN000009886). The study was approved by local institutional review boards and/or ethics committees and conducted in accordance with good clinical practice guidelines and the declaration of Helsinki. All patients provided written informed consent.

Patients with recurrent HER2-negative breast cancer who had received AT regimens in previous treatment (either neoadjuvant/adjuvant or first-line treatment) were eligible for this phase II study. Eligible patients had received no or only one prior line of chemotherapy for recurrent disease and had or had not received endocrine therapy. Other inclusion criteria included: female patients with a histological diagnosis of invasive breast cancer; ECOG performance status (PS) 0–1; measurable disease by RECIST; an interval of at least 6 months after the end of prior anthracycline and taxane-based chemotherapy; no prior use of eribulin; adequate organ function within 14 days before enrollment (neutrophil count >  = 1,500/mm^3^, platelet count >  = 1,00,000 mm^3^, hemoglobin >  = 9.0 g/dL, total bilirubin <  = 2.0 mg/dL, AST (GOT) and ALT (GPT) < 100 IU/L (< 150 IU/L for patients with hepatic metastasis), serum creatinine <  = 1.5 mg/dL); and no clinical abnormalities on electrocardiography.

Exclusion criteria were: active infection or fever suggestive of infection; history of serious drug allergy; severe renal or hepatic impairment (jaundice); interstitial pneumonia or pulmonary fibrosis evident on chest radiograph: large amounts of pleural effusion or ascites requiring drainage; uncontrolled hypertension or diabetes; chronic systemic (oral or intravenous) steroid therapy; pregnant women or women of child-bearing potential; active other malignancy; history of clinically significant mental disorder or central nervous system damage; active brain metastases; concurrent participation in other therapeutic clinical studies; and patients considered unsuitable for study participation by the investigator for any reasons (e.g., rapid disease progression necessitating immediate achievement of response to avoid a life-threatening condition).

Eligible patients were registered and randomized by the central registration office. Investigators at enrolling centers were notified of the treatment allocation for each patient. Patients were randomly allocated by computer to receive eribulin monotherapy (arm A) or TPC monotherapy (arm B) at a ratio of 1:1 using minimization method with stratification by hormone receptor status (positive versus negative), disease-free interval (1 year or more versus less than 1 year) and treatment line (first versus second). In arm B, TPC was selected from four existing microtubule inhibitors (paclitaxel, docetaxel, nab-paclitaxel and vinorelbine).

The primary endpoint of the study was PFS as assessed using RECIST (Version 1.1). Secondary endpoints included TTF, overall response rate (ORR), response duration, and frequency of adverse events. TTF was selected because, in addition to efficacy, it is also important in recurrent breast cancer that treatment can be continued without impairing the quality of life.

In previous phase II studies, PFS of advanced recurrent breast cancer patients treated with eribulin was around 3–6 months [9]. In the phase III EMBRACE study in previously treated patients, PFS was 3.7 months in eribulin group and 2.2 months in TPC group [7]. Based on these observations, we hypothesized that in the present study involving AT-pretreated patients, median PFS would be 7 months in eribulin group compared with 4 months in TPC group. Under the assumption that PFS follows an exponential distribution, this 3-month improvement with eribulin corresponds to a risk reduction of 40% (hazard ratio 0.57).

Assuming an entry period of 12 months and a follow-up period of 6 months, at least 39 patients per group, 78 in total, were required to detect this reduction with a two-sided *α* of 0.20 and a power of 80% [10]. Considering the expected small number of ineligible patients and others excluded from the analysis, 40 patients per group and 80 in total were planned to be enrolled.

In arm A, eribulin (1.4 mg/m^2^) was administered on day 1 and 8 every 21 days. Expected significant adverse effects of eribulin include bone marrow suppression, infections, hepatic dysfunction and interstitial pneumonia. To avoid the risk of these events, treatment was discontinued or delayed if patients failed to meet all of the following criteria before each cycle: (1) neutrophil count >  = 1,000 mm^3^, (2) platelet count >  = 75,000 mm^3^, (3) hemoglobin >  = 9.0 g/dL, (4) total bilirubin <  = 2.0 mg/dL, (5) AST (GOT) and ALT (GPT) < 100 IU/L (< 150 IU/L for patients with hepatic metastasis), and (6) serum creatinine <  = 1.5 mg/dL. If any of the following adverse events occurred, the dose or dosing schedule was modified at the discretion of the investigator: (1) grade >  = 3 neutropenia with a fever over 38.0 °C, (2) thrombocytopenia with a platelet count of < 25,000 mm^3^, with bleeding, or requiring blood transfusion, or (3) grade >  = 3 non-hematological toxicities. Typically, dose modification for eribulin was made from the initial dose of 1.4 mg/m^2^ to 1.1 mg/m^2^ and then to 0.7 mg/m^2^.

In arm B, TPC (monotherapy with one of four existing microtubule inhibitors: paclitaxel, docetaxel, nab-paclitaxel and vinorelbine) was administered according to the standard dosing schedule described in the package insert as well as information on the proper use of each drug (paclitaxel: 80 mg/m^2^ on day 1, 8, 15 every 28 days or 175 mg/m^2^ on day 1 every 21 days, docetaxel: 60 mg/m^2^ on day 1 every 21 days, nab-paclitaxel: 260 mg/m^2^ on day 1 every 21 days vinorelbine: 25 mg/m^2^ on days 1 and 8 every 21 days). Modifications in dose or dosing schedule were allowed according to described in the package insert as well as information on the proper use of each drug considering the patient’s condition and other reasons.

Tumors were imaged at baseline and every 12 weeks during treatment and evaluated for change in size from baseline. Treatment was continued until disease progression or unacceptable toxicity occurred.

At baseline, demographic and adverse event data were collected and recorded in case report forms. During the protocol treatment, adverse events (subjective and objective signs and symptoms) were assessed before each cycle. Reported events were graded according to the Common Terminology Criteria for Adverse Events, Version 4.0, Japanese edition, Japan Clinical Oncology Group version. Event term, onset date, outcome, date of outcome (resolution), grade, seriousness, relationship to the study treatment, and action taken by the investigator/sub-investigator were recorded in case report forms. After the protocol treatment, adverse events were monitored for 1 year or until discontinuation of follow-up and assessed every 6 months.

During the study, we calculated the Bayesian posterior probability that eribulin would be found superior, i.e., the hazard ratio for PFS would be lower than one, and early termination of the study was to be considered if the probability was less than 5%. Efficacy was analyzed for all eligible patients who received > = 1 cycle on a per-protocol basis and safety analyses were performed on all eligible patients who received at least one dose of study treatment. For PFS, TTF and response duration, the Kaplan–Meier method was used to estimate survival curves, median times and two-sided 95% confidence intervals. ORR was calculated as the percentage of eligible patients with measurable disease who achieved a complete or partial response according to RECIST Version 1.1. All statistical tests were two sided and performed at a significance level of *α* = 0.05. Missing values were not imputed and outliers or extreme values were not removed from analyses. All analyses, such as the log-rank test, the chi-square test and others, were performed using the SPSS software, version 22.0 for windows. For adverse events, the grade distribution was analyzed and the incidence by grade was calculated.

## Results

**Fig. 1:**
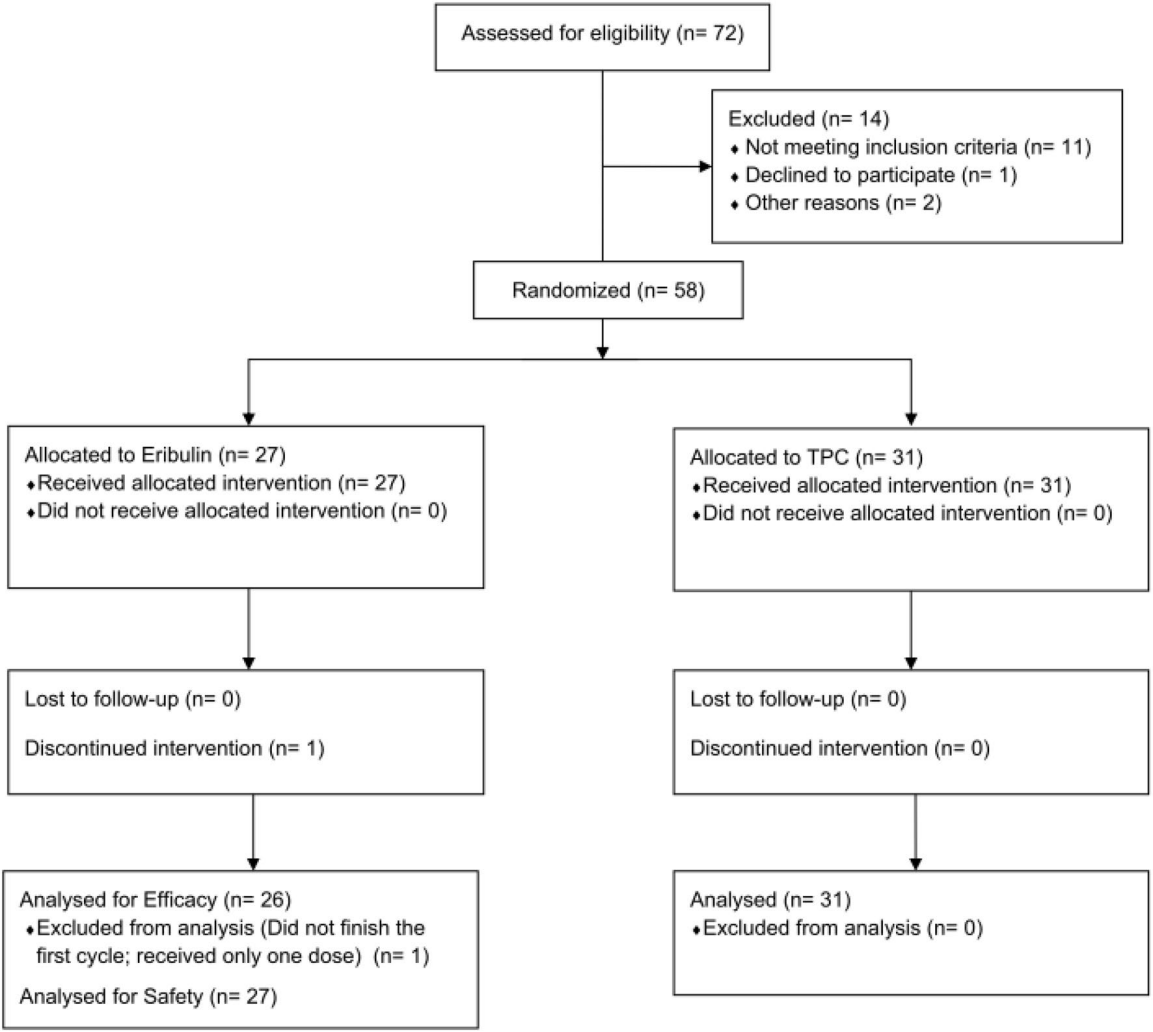
CONSORT flow diagram

From May 2013 to January 2017, 72 were assessed for eligibility for the study. As of data cut-off of 18 Oct 2017, the median follow-up was 14.0 months. A patient flow diagram is shown in Fig. 1. After excluding 14 patients (including 11 who were ineligible), 58 enrolled and randomized in the study. All of the 58 randomized patients were included in the full analysis set. The per-protocol set comprised 57 patients, excluding one patient in eribulin group who received the first dose but discontinued the study for personal reasons before completing the first cycle.

Demographic and clinical characteristics of the 57 patients in the per-protocol set are shown in Table 1. Treatment compliances were retained in two arms.

**Table 1:** Patients characteristics

	Overall (*n* = 57)	Eribulin (*n* = 26)	TPC (*n* = 31)
Age, median (range)	58.0 (33–82) *n* (%)	56.5 (39–82) *n* (%)	58.0 (33–74) *n* (%)
ER			
Positive	43 (75.4)	20 (76.9)	23 (74.2)
Negative	13 (22.8)	5 (19.2)	8 (25.8)
Unknown	1 (1.8)	1 (3.8)	0 (0.0)
PgR			
Positive	29 (50.9)	14 (53.8)	15 (48.4)
Negative	27 (47.4)	11 (42.3)	16 (51.6)
Unknown	1 (1.8)	1 (3.8)	0 (0.0)
Disease-free interval			
1 year >	11 (19.3)	3 (11.5)	8 (25.8)
1 year ≤	46 (80.7)	23 (88.5)	23 (74.2)
Treatment line			
1st line	37 (64.9)	19 (73.1)	18 (58.1)
2nd line	20 (35.1)	7 (26.9)	13 (41.9)
Menopausal status			
Pre-menopausal	14 (24.6)	6 (23.1)	8 (25.8)
Post-menopausal	40 (70.2)	18 (69.2)	22 (71.0)
Unknown	3 (5.3)	2 (7.7)	1 (3.2)

*TPC* treatment of physician’s choice, *ER* estrogen receptor, *PgR* progesterone receptor

The median age was 58 years (range, 33–82 years) and 43 patients (75.4%) were ER positive. Study therapy was given as first-line treatment in 38 patients (64.9%) and as second-line treatment in 20 (35.1%). TPC group included 24 patients treated with vinorelbine, six patients with paclitaxel and one patient with docetaxel.

**Fig. 2:**
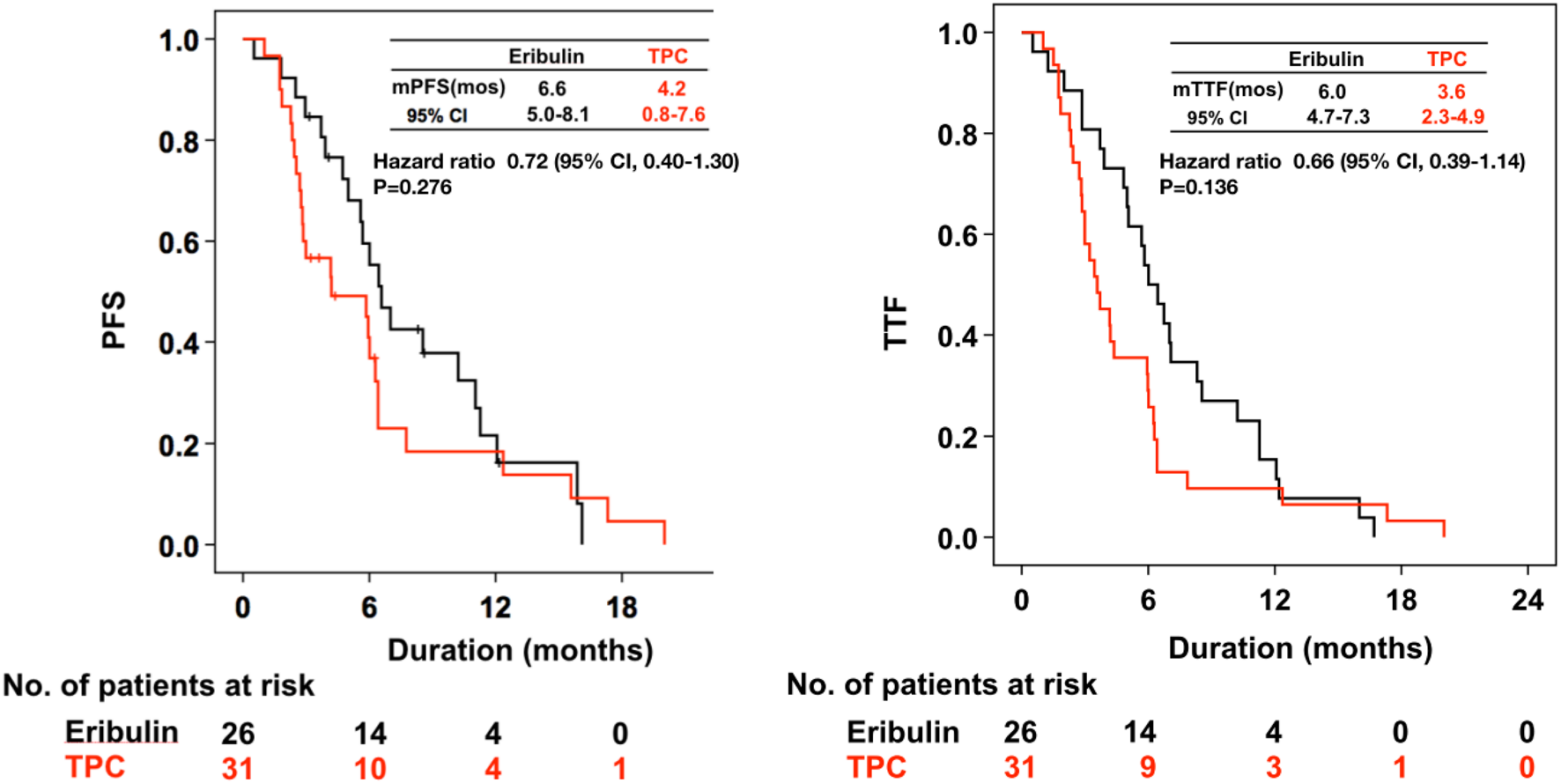
Results of PFS and TTF in eribulin and TPC group

Results for PFS, TTF, ORR and duration of response are shown in Table 2 and Fig. 2. Median PFS was 6.6 months (95% CI, 5.0–8.1 months) with eribulin compared with 4.2 months (95% CI, 0.8–7.6 months) with TPC (hazard ratio 0.72 [95% CI, 0.40–1.30], *p* = 0.276). Median TTF was 6.0 months (95% CI, 4.7–7.3 months) with eribulin and 3.6 months (95% CI, 2.3–4.9 months) with TPC (hazard ratio 0.66 [95% CI, 0.39–1.14], *p* = 0.136). ORR was 19.2% with eribulin and 19.4% with TPC. But eribulin showed higher stable disease rate as of 61.5% and lower progressive disease rate as of 19.2% compared to those of TPC as of 35.5 and 41.9% respectively.

**Table 2:** PFS, TTF and response

		Eribulin (*n* = 26)	TPC (*n* = 31)	*p* value
Progression-free survival	Median (month)	6.6 (5.0–8.1)	4.2 (0.8–7.6)	0.273
Time to treatment failure	Median (month)	6.0 (4.7–7.3)	3.6 (2.3–4.9)	0.131
Tumor response	Complete response	0 (0.0%)	0 (0.0%)	
	Partial response	5 (19.2%)	6 (19.4%)	
	Stable disease	16 (61.5%)	11 (35.5%)	
	Progressive disease	5 (19.2%)	13 (41.9%)	
	Unknown	0 (0.0%)	1 (0.3%)	
Overall response rate		5 (19.2%)	6 (19.4%)	0.190
Duration of response	Median (month)	3.0 (2.1–3.9)	2.8 (2.4–3.3)	0.794

*PFS* progression-free survival, *TTF* time to treatment failure, *TPC* treatment of physician’s choice

Table 3 summarizes adverse events reported in the study. The most common AEs in all grades with eribulin were neutropenia (33.3%), leukopenia (18.5%), neuropathy (14.8%) and alopecia (7.4%). In TPC, the most common AEs were neutropenia (22.6%), leukopenia (3.2%), neuropathy (6.5%) and alopecia (6.5%). The incidence of sensory neuropathy was low in both groups. The most common grade three or worse adverse event was neutropenia, which occurred in six of 27 (22.2%) and five of 31 (16.1%) patients receiving eribulin and TPC, respectively. Febrile neutropenia was reported as of 3.7% with eribulin and 3.2% with TPC.

**Table 3:** Adverse events

AE (%)	Eribulin (*n* = 27)		TPC (*n* = 31)	
	All grades	All grades	All grades	Grade 3/4
Hematologic events				
Neutropenia	33.3	22.2	22.6	16.1
Leukopenia	18.5	3.3	9.7	3.2
Febrile neutropenia	3.7	3.7	3.2	3.2
Anemia	0.0	0.0	6.5	0.0
Non-hematologic events				
Neuropathy	14.8	0.0	6.5	0.0
Alopecia	7.4	0.0	6.5	0.0
Pharyngitis	7.4	0.0	3.2	0.0
Fracture	7.4	0.0	0.0	0.0
Anorexia	7.4	0.0	0.0	0.0
Fatigue	3.7	0.0	6.5	3.2
Malaise	3.7	0.0	3.2	3.2
Dysgeusia	3.7	0.0	3.2	0.0
Nausea	3.7	0.0	0.0	0.0
Gastritis	3.7	0.0	0.0	0.0
Diarrhea	3.7	0.0	0.0	0.0
Mucositis oral	3.7	0.0	0.0	0.0
Hypertension	3.7	0.0	0.0	0.0
Chest pain	3.7	0.0	0.0	0.0
ALT/AST increase	0.0	0.0	9.7	9.7
Arthritis	0.0	0.0	3.2	0.0
Myalgia	0.0	0.0	3.2	0.0
Dyspnea	0.0	0.0	3.2	0.0
Edema	0.0	0.0	3.2	0.0
Injection site reaction	0.0	0.0	3.2	0.0

*TPC* treatment of physician’s choice

## Discussion

Anthracycline or taxane-based chemotherapy regimens have been used in the first-line treatment of advanced recurrent breast cancer. These include AC (doxorubicin + cyclophosphamide), CAF (cyclophosphamide + doxorubicin + 5-FU), CMF (cyclophosphamide + methotrexate + 5-FU) and AT and there are some reports suggesting that anthracycline-containing regimens were more effective than anthracycline-free regimen, such as CMF [11–13]. In patients who have developed recurrent disease after neoadjuvant/adjuvant AT therapy, regimens other than AT are usually selected. Taxanes not used in previous treatment, non-taxane microtubule inhibitors and oral fluoropyrimidines are often used but no standard strategy has been established.

The present study was a randomized phase II study compared the efficacy and safety of eribulin versus TPC as first- or second-line treatment for recurrent HER2-negative breast cancer in patients previously received AT, to determine whether to proceed to a phase III study to confirm the superiority of eribulin over standard treatment in the same design. As the study did not meet the primary endpoint (PFS) statistically, we decided not to move on to phase III. However, patients in the eribulin group had longer PFS and TTF numerically comparing to those in TPC group. Eribulin also showed numerically higher stable disease rate and lower progressive disease rate compared to those of TPC.

Previous studies have reported promising results for eribulin. In a multicenter, single-arm, phase II study evaluating the use of eribulin as first-line therapy in 56 patients with locally recurrent or metastatic HER2-negative breast cancer (study 206), ORR was high at 29% with a clinical benefit rate of 52% and median duration of response was 5.8 months [14] A pooled analysis found that eribulin improved OS of various patient subgroups with advanced/metastatic breast cancer who had previously received an anthracycline and a taxane, especially in those with HER2-negative disease [2]. A phase III comparative study (study 305) reported that eribulin improved OS of patients with previously treated metastatic breast cancer compared with TPC [15]. In Japan, an observational study concluded that eribulin may be a first-line treatment candidate for patients with HER2-negative advanced breast cancer [16]. The real-world observational ESME program enrolling 16,703 metastatic breast cancer patients evaluated outcomes of patients treated with eribulin as second-, third- and fourth-line treatment and found a significant improvement in OS and PFS compared with other chemotherapies for all lines among the HER2-negative patient subgroup [17]. Another real-world study also reported that eribulin was effective in heavily pretreated metastatic breast cancer [18]. Additional studies are ongoing and results are awaited, including a phase III study comparing eribulin versus paclitaxel in HER2-negative metastatic breast cancer patients who have received no or one prior chemotherapy regimen (ACCRU study) [19]. This study is important because there have been only a limited number of such studies worldwide directly comparing eribulin and standard treatment.

As for the safety, most adverse events were grade one or two and serious AEs and life-threatening AEs were not reported in both arms, respectively. Both Arm treatments had manageable safety profiles consistent with their known adverse effects. Eribulin therapy was well tolerated in the present study, with only 14.8% of patients experiencing neuropathy of any grade. Toxicity profile was similar to that in previous studies. For example, in a Japanese phase II single-arm study, adverse events were observed in all 81 patients included in the safety analysis and the most common adverse events were neutropenia (98.8%), leukopenia (98.8%), lymphopenia (54.3%) and alopecia (58.0%) [8]. The reported incidences of peripheral sensory neuropathy of any grade were relatively low at 21.0% in this study and 12.3% in the phase III EMBRACE study, compared with 60–70% reported with taxanes [20]. In one report, the incidence of grade 4 toxicity in a real-world setting was lower than that reported in previous clinical studies [21].

The limitations of this study include the small sample size due to the preliminary nature of phase II studies. This study was also underpowered by the higher-than-expected number of ineligible patients. Therefore, it is still possible that eribulin may be more useful than standard treatment. It will be a future task to narrow down the target patients using a certain marker and identify the eribulin-effective group.

In conclusion, eribulin did not improve PFS statistically compared with TPC as first- or second-line treatment for recurrent HER2-negative BC following AT-based chemotherapy in this randomized phase II study. Considering the longer PFS and TTF numerically in the eribulin group, further validation studies are needed.
