# Correction to: Clinical usefulness of eribulin as first- or second-line chemotherapy for recurrent HER2-negative breast cancer: a randomized phase II study (JBCRG-19)

**DOI:** 10.1007/s10147-021-01940-w

**Published:** 2021-05-27

**Authors:** Kenjiro Aogi, Kenichi Watanabe, Masahiro Kitada, Takafumi Sangai, Shoichiro Ohtani, Tomoyuki Aruga, Hidetoshi Kawagichi, Tomomi Fujisawa, Shigeto Maeda, Takashi Morimoto, Nobuaki Sato, Shintaro Takao, Satoshi Morita, Norikazu Masuda, Masakazu Toi, Shinji Ohno

**Affiliations:** 1 Department of Breast Oncology, National Hospital Organization Shikoku Cancer Center, Kou 160, Minamiumemoto-machi, Matsuyama, Ehime, 791-0280 Japan; 2 Department of Breast Surgery, National Hospital Organization Hokkaido Cancer Center, Sapporo, Japan; 3 Breast Disease Center, Asahikawa Medical University Hospital, Asahikawa, Japan; 4 Department of Breast and Thyroid Surgery, Chiba University Hospital, Chiba, Japan; 5 Department of Breast Surgery, Hiroshima City Hiroshima Citizens Hospital, Hiroshima, Japan; 6 Department of Breast Surgery, Tokyo Metropolitan Cancer and Infectious Diseases Center Komagome Hospital, Tokyo, Japan; 7 Department of Breast Surgery, Matsuyama Red Cross Hospital, Matsuyama, Japan; 8 Department of Breast Oncology, Gunma Prefectural Cancer Center, Ohta, Japan; 9 Department of Surgery, National Hospital Organization Nagasaki Medical Center, Nagasaki, Japan; 10 Department of Breast Surgery, Yao Municipal Hospital, Osaka, Japan; 11 Department of Breast Oncology, Niigata Cancer Center Hospital, Niigata, Japan; 12 Department of Breast Surgery, Hyogo Cancer Center Hospital, Kobe, Japan; 13 Department of Biomedical Statistics and Bioinformatics, Graduate School of Medicine, Kyoto University, Kyoto, Japan; 14 Department of Surgery, Breast Oncology, National Hospital Organization Osaka National Hospital, Osaka, Japan; 15 Department of Breast Surgery, Graduate School of Medicine, Kyoto University, Kyoto, Japan; 16 Breast Oncology Center, The Cancer Institute Hospital of Japanese Foundation for Cancer Research, Tokyo, Japan

## Correction to: International Journal of Clinical Oncology 10.1007/s10147-021-01920-0

In the original publication, the name of the fourth author should be: Takafumi Sangai and his affiliation should be:

Department of Breast and Thyroid Surgery, Chiba University Hospital, Chiba, Japan

The corrected name and affiliation are given in this correction.

