# Correction to: Clinical usefulness of eribulin as first- or second-line chemotherapy for recurrent HER2-negative breast cancer: a randomized phase II study (JBCRG-19)

**DOI:** 10.1007/s10147-022-02237-2

**Published:** 2022-09-27

**Authors:** Kenjiro Aogi, Kenichi Watanabe, Masahiro Kitada, Takafumi Sangai, Shoichiro Ohtani, Tomoyuki Aruga, Hidetoshi Kawaguchi, Tomomi Fujisawa, Shigeto Maeda, Takashi Morimoto, Nobuaki Sato, Shintaro Takao, Satoshi Morita, Norikazu Masuda, Masakazu Toi, Shinji Ohno

**Affiliations:** 1 Department of Breast Oncology, National Hospital Organization Shikoku Cancer Center, Kou 160, Minamiumemoto-machi, Matsuyama, Ehime, 791-0280 Japan; 2 Department of Breast Surgery, National Hospital Organization Hokkaido Cancer Center, Sapporo, Japan; 3 Breast Disease Center, Asahikawa Medical University Hospital, Asahikawa, Japan; 4 Department of Breast Thyroid Surgery, Kitasato University Hospital, Sagamihara, Japan; 5 Department of Breast Surgery, Hiroshima City Hiroshima Citizens Hospital, Hiroshima, Japan; 6 Department of Breast Surgery, Tokyo Metropolitan Cancer and Infectious Diseases Center Komagome Hospital, Tokyo, Japan; 7 Department of Breast Surgery, Matsuyama Red Cross Hospital, Matsuyama, Japan; 8 Department of Breast Oncology, Gunma Prefectural Cancer Center, Ohta, Japan; 9 Department of Surgery, National Hospital Organization Nagasaki Medical Center, Nagasaki, Japan; 10 Department of Breast Surgery, Yao Municipal Hospital, Osaka, Japan; 11 Department of Breast Oncology, Niigata Cancer Center Hospital, Niigata, Japan; 12 Department of Breast Surgery, Hyogo Cancer Center Hospital, Kobe, Japan; 13 Department of Biomedical Statistics and Bioinformatics, Graduate School of Medicine, Kyoto University, Kyoto, Japan; 14 Department of Surgery, Breast Oncology, National Hospital Organization Osaka National Hospital, Osaka, Japan; 15 Department of Breast Surgery, Graduate School of Medicine, Kyoto University, Kyoto, Japan; 16 Breast Oncology Center, The Cancer Institute Hospital of Japanese Foundation for Cancer Research, Tokyo, Japan

## Correction to: International Journal of Clinical Oncology (2021) 26:1229–1236 10.1007/s10147-021-01920-0

In the original publication, the name of fourth and seventh authors were incorrectly published as: Takashi Sangai and Hidetoshi Kawagichi. The correct version of names are: Takafumi Sangai and Hidetoshi Kawaguchi as given in this Correction.

The original publication has been corrected.

